# Detection of Homologous Recombination Events in Bacterial Genomes

**DOI:** 10.1371/journal.pone.0075230

**Published:** 2013-10-07

**Authors:** Wei-Bung Wang, Tao Jiang, Shea Gardner

**Affiliations:** 1 Department of Computer Science, University of California Riverside, Riverside, California, United States of America; 2 Lawrence Livermore National Laboratory, Livermore, California, United States of America; University of North Carolina at Charlotte, United States of America

## Abstract

We study the detection of mutations, sequencing errors, and homologous recombination events (HREs) in a set of closely related microbial genomes. We base the model on single nucleotide polymorphisms (SNPs) and break the genomes into blocks to handle the rearrangement problem. Then we apply a dynamic programming algorithm to model whether changes within each block are likely a result of mutations, sequencing errors, or HREs. Results from simulation experiments show that we can detect 31%–61% of HREs and the precision of our detection is about 48%–90% depending on the rates of mutation and missing data. The HREfinder software for predicting HREs in a set of whole genomes is available as open source (http://sourceforge.net/projects/hrefinder/).

## Introduction

Phylogenetic trees are commonly used to represent the evolutionary history of a set of extant species in biology. If all organisms only inherit their genetic materials vertically, i.e., from their parents, then the tree representation would be sufficient. However, there is evidence that organisms may get genetic materials from organisms other than their parents [1]–[3], and this process is called *homologous recombination event* (HRE). An *HRE* is caused by a homologous recombination, in which the incoming DNA molecules are highly similar to those in the recipient genome. HREs may cause the incongruence between gene trees drawn by different genes, and may lead to inaccurate construction of phylogenetic trees [4]. Detection of HREs will help construct a more accurate phylogenetic network [5].

To detect HREs, a standard approach is to compare the gene trees and the species tree, construct the reconciled tree and detect the HREs (e.g. [6], [7]). These methods do not use the whole-genome information, and do not utilize the gene positional information. Methods based on alignments (e.g. [8]–[10]) use the positional information and have a higher accuracy. The main drawback of the alignment approach is poor scalability when dealing with the whole genomes of dozens of bacterial strains. Most researchers would choose to align only a few target genomes/genes instead of many whole genomes. A small subset of genes risk poor phylogenetic inference if the genes are involved in HREs [4]. If the species tree is drawn by selecting large numbers of characters that are distributed across the genomes, the influence of recombined single genomic regions in tree topology will be diminished, resulting in a tree that reflects the evolutionary history of the majority of the genomes [3] and helps detect the homoplastic changes, those that conflict with the evolutionary pattern captured by the tree, may be more parsimoniously explained by HREs than by mutations and sequencing errors. Convergent evolution could be erroneously classified as HRE by our software, as a single HRE may more parsimoniously explain a cluster of similar SNPs than multiple parallel mutations in the same genome region among disparate strains.

In this paper, we study the detection of mutations, HREs and sequencing errors given the SNPs and SNP positions of a set of closely related strains with an evolutionary species tree. The SNPs of all leaf nodes are mostly known with some missing, but the SNPs of all internal nodes are unknown. Some known SNPs might be incorrect because of sequencing errors. Some genomes might be in the form of contigs, i.e., the SNP positions are only in the correct order and orientation within a contig. We want to reconstruct the SNPs of internal nodes with regard to 3 possible events. (1) Mutations. A single SNP may change when an internal node passes its SNPs to its child node. (2) HREs. A node may get a segment of SNPs from any other node which is not one of its descendants. (3) Sequencing errors. The data we have may be wrong.

**Figure 1:**
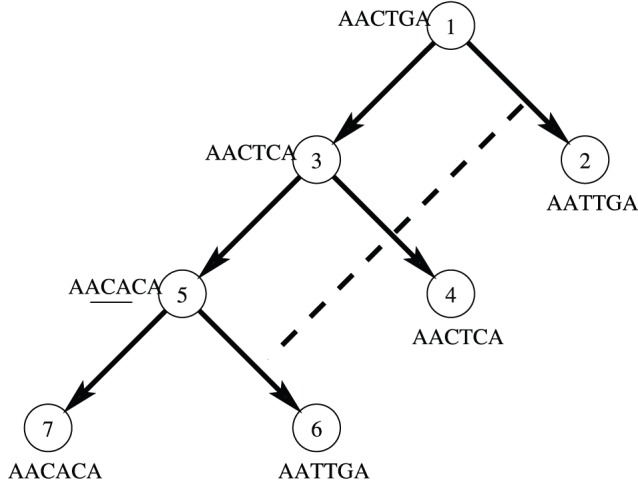
An example of detection of HREs. The SNPs on node 6 are better explained by an HRE from node 2 than inheritance from node 5 with three mutations. The first 2 A’s and last A do not represent SNPs, but merely serve as sequence context for the SNPs in between.

We cannot distinguish sequencing errors from mutations that occur on the leaf nodes. For simplicity, all SNP disagreements between a leaf node and its parent node are considered as “errors” (although in reality some may be true SNP variations). Therefore, mutations refer to SNP changes at internal nodes, and errors refer to SNP changes at leaf nodes. Each event has a weight. The weights of mutation/HRE/error are 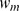, 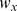, and 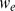, respectively. We want to reconstruct the events and SNPs of all nodes (including leaf nodes because there might be errors), while minimizing the total weight. The frequencies of mutation/HRE/error events are low, and the assignment that minimizes the total weight would give a reasonable explanation [3]. Note that the error weight 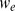 is always less than the mutation weight 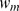, since SNP variations on leaf nodes are always considered to be errors. Considering a homologous recombination event, if the source or the destination mutate in the sequence context around the SNP, then the SNP locus from the donor appears to be missing in the receiver, or vice versa. Inversions that occur after an HRE and whose endpoints fall within the HRE region also disrupt the co-linearity of SNP loci across genomes. Therefore, we only consider HREs that have the same SNP loci in the same order and orientation in both the source and destination (with some exceptions explained in Section 2.1), although differences from mutations/errors are allowed between donor and recipient. We use a greedy algorithm to partition genomes into *blocks* in which inversions do not take place. We then use the dynamic programming technique to assign mutations/HREs/errors in each block. We also consider possible HREs from an out-group, i.e., some species not in the given evolutionary species tree. If a genome has a many SNPs alleles that differ from other genomes in the tree within a small segment of adjacent SNP loci, then we consider assigning an HRE from an out-group to this segment (see Section 2.3). Figure 1 shows an example of how HREs can leave evidence within a block. There are six SNPs loci, and the SNPs on the leaf nodes (2, 4, 6, 7) are known. We can explain SNPs on node 6 by three mutations or one HRE, and we assume that one HRE is more likely than three neighboring errors and set the weights accordingly (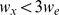).

We have implemented our algorithms that partition genomes into blocks and assign mutations/HREs/errors. We have tested the program on both simulated data and real data. The experimental simulation results demonstrate that there are many HREs and mutation events that leave no evidence to be detected, and the detection accuracy mainly depends on the mutation rate, HRE rate, and the size of the evolutionary tree.

## Methods

The sequences of source and destination of a HRE should be similar, i.e., there should be the same set of SNPs in the same order and orientation in the HRE regions of both donor and recipient genomes. However, the SNP order/orientation may not be identical all genomes, because of genome rearrangement events, i.e., inversions and transpositions, and we have to focus on regions in which all genomes have the same SNP order and orientation. A *locally collinear block* is a homologous region of sequence shared by two or more of the genomes under study, and does not contain any rearrangements of homologous sequence [11]. In this paper, we simply use *blocks* to refer locally collinear blocks. SNPs in a block should be in the same order across all genomes, with some exceptions explained in Section 2.1.

We first partition the genomes into blocks by a greedy block extension algorithm, then we consider each block separately. Within each block, for each SNP locus, we use dynamic programming to reconstruct the SNPs of internal nodes in the evolutionary tree with the minimum number of mutations. Then within each block, we check if we can assign HREs to further reduce the total weight by dynamic programming. We also consider possible HREs from an out-group not in the input genomes. After assigning mutations/HREs/errors from within the tree, we trace the origin of each SNP allele and evaluate if there is any evidence indicating HRE from an out-group. Note that these steps represent only one reasonable approach to this problem, and optimal solution for each step does not guarantee optimal solution at the end.

### 2.1 Computing Blocks with Duplications and Missing SNPs

Considering a possible block 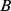 and a genome 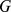, we say that 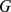 *agrees* with 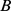 if, given the genome 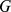, there is no evidence that suggests an inversion within the block 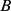. A straightforward example is, if all the SNPs in 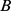 appear consecutively in 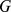 in the same order and orientation, or all in the reverse order and complement orientation, then 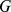 agrees with 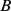. Different orders usually suggest inversions, but there are some exceptions.

1. Missing. A SNP may appear in 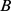 but be absent in 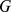, and it does not suggest an inversion. For example, 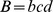, 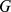 contains a SNP sequence 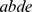 and 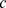 is absent in 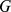, then 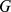 should agree with 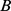.
2. Duplication. There might be duplicated SNPs inserted in 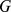 and they could alter the SNP order. For example, 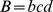, 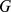 contains a SNP sequence 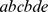, then the second 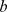 in 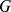 should be considered as a duplicated SNP, and 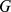 should agree with 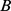.
3. Contigs. The genome may be in contig form, which makes the SNP order in 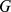 unclear. For example, 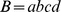, 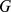 contains a contig ending with SNP sequence 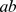 and a contig starting with 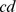, then 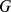 should agree with 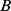.

We formally define the notion of agreement as follows.

#### Definition 1

*Let 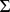 be the set of forward and reverse complement of all SNPs. A block is a string 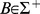 and a genome is a set of strings 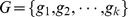, 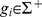 (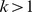 if in contig form). Let 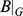 be the subsequence of 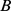 obtained by deleting SNPs absent in 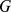. Let 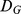 be the set of SNPs that appear more than once in 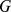. We say the genome 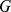 agrees with the block 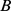 if and only if there exists a string 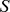 such that the two following statements both hold. (1) There exists a concatenation 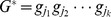 allowing reverse complement and 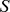 is a substring of 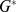. (2) 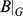 is a subsequence of 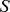 and 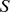 can be obtained from 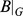 by inserting only SNPs in 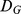.*

When considering if a genome 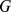 agrees with a block 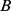, we try to match the SNP order and orientation in 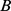 and 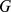. If a SNP 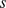 appears in 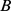 but does not appear in 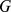, then 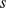 should be skipped in 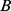 in the matching. If a SNP 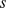 appears in 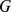 more than once, then we can choose to skip 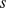 in 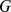 or not, based on if it makes the SNP order/orientation in 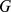 different from those in 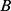. When we try to match the SNP order/orientation but the comparison reaches the end of a contig, then the next match in 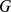 can start from any other end of a contig. Let 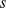 be the SNP in 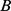 we want to match when the comparison reaches the end of a contig in 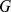. We check all occurrences of 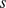 and see if any occurrence of 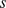 is at the end of a contig (or only duplicated SNPs between 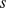 and the end of the contig) and if the occurrence of 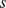 is in the correct orientation. If there is a such occurrence, we can keep matching from the occurrence. If there are multiple such occurrences, then there are multiple ways to match 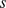 and we have to enumerate and check all possibilities. We call this a *jump-over-contig* step.

We try to explain all genomes with the minimum number of inversion endpoints, i.e., as few blocks as possible. We use a greedy block extension algorithm so that every block is maximal, and minimize the number of blocks. The block extension algorithm works as follows. A block starts from a single SNP. Each round we try to extend a block 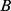, we pick a SNP 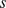 which is next to 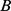 in some genome, and test if all other genomes agree with the new block candidate 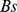. If all genomes agree with 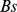, then we extend 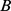 to 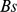 and start the next round. If there is any genome that does not agree with 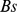, then we pick up another SNP 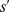 which is next to 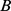 in some genome. If there is no such SNP that extends 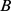 in either forward or reverse direction, then we stop extending and output 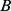 as a block. Tables 1 and 2 outline the main idea of the algorithm.

**Table 1:** Algorithm GetBlock.

1: 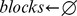 2: **for each** genome do3: **for** **each** SNP 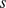 do4: **if** 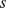 has not been included by any block **then**5: 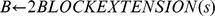 6: mark all SNPs in 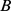 as included7: 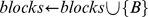 8: **return** 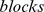

**Table 2:** Algorithm BlockExtension 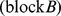.

1: **for** both forward and reverse direction do /*reverse B when needed */2: **for each** genome 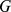 **do**3: Let 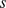 be the next SNP after the block 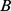 in genome 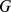 4: **if** 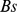 has not been tested **then**5: **if** all genomes agree with 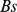 **then**6: 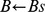

The time complexity of the algorithm is determined by how fast we can determine if a genome agrees with a block. Assume 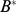 is returned by Algorithm 2 and there is no duplication, then a straightforward implementation will take 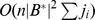 time, where 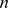 is the number of genomes, 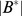 is the length of the block, and 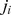 is the product of all jump-over-contig enumerations on genome 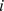. Note that duplications make it possible that a genome may agree with a small block in multiple ways in our algorithm, which theoretically increases the time complexity, and complicates the optimization. We choose not to optimize the implementation because our experiments show that a straightforward implementation yields a reasonable running time. For example, it takes 2 minutes for the *Bulkhorderia pseudomallei* dataset with 122 thousand SNPs and 26 strains. This is because duplications and jump-over-contigs do not occur very frequently.

In our algorithm, if a SNP 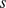 is absent in a genome 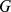, then 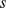 will never make 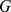 disagree with a block. If 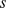 is next to a inversion endpoint, then 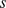 may appear in two different blocks. For example, genome 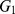 has a SNP sequence 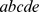 and genome 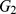 has 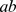 and 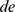 but 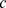 is absent in 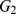. Our algorithm will produce two blocks 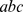 and 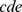, and we say these two blocks *overlap*. Duplications may also create overlapping blocks. For example, 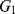 has SNP sequence 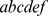 and 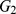 has 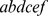 and 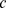, 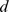 elsewhere. Our algorithm will get two blocks 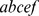 and 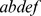. Therefore, after getting blocks, the summation of number of SNPs in all blocks, denoted as *increased* number of SNPs, is usually much more than the number of given SNPs. Note that overlapping blocks may result in duplicated HREs at the end, and we may overcount the number of HREs. However, we simply accept overcounting since our objective is to find HREs, not to count HREs.

### 2.2 Inside of a Block with no Inversions

We now consider a single block, and the corresponding SNPs of the block in all genomes. Our objective is to reconstruct the history of the block on each node of the evolutionary tree. The SNP order of the block should be the same in all genomes but there might be missing SNPs. For each SNP locus, we reconstruct the SNPs of internal nodes assuming there are only mutations and errors, and minimize the total weight of mutations (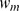) and errors (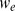) at the same time. This is a weighted small parsimony problem and can be solved by dynamic programming in linear time [12].

After inferring the SNPs of the internal nodes, we then compute if we can assign HREs. Let 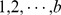 be the SNP indices of the block we consider. For each internal node 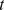 as a possible HRE destination, we define 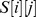 as the minimum total weight considering SNPs 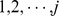 assuming node 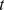 inherits SNP 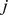 from node 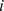. Let 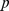 be the parent node of 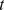, 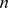 the number of nodes, and 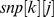 SNP 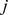 of node 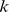. We derive the recurrence relations for 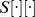: (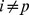).
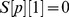
(1)

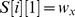
(2)

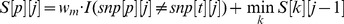
(3)

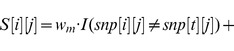
(4)



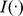
 is the indicator function in the above equations. Equation (3) represents the case that SNP 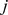 is not from an HRE, and Equation (4) represents the case that SNP 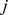 is extending an existing HRE (top option in bracket) or starting a new HRE (bottom option in bracket). In Equation (3) and (4), 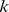 is enumerated from all possible source nodes, i.e., all other nodes that are not descendants of node 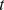. We charge the weight of an HRE at the beginning of the HRE (Equation (4)), but do not charge at the end (Equation (3)). Note that Equation (4) also allows us to have mutations on a segment of HRE. For the leaf nodes, the recurrence relations are identical except each 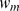 is replaced by 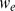. With the recurrence relations established, a standard dynamic programming technique with backtracking would be sufficient to assign mutations/HREs optimally [12], [13]. There are 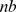 entries in 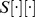, and it takes 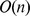 time to compute each entry. The time complexity is 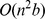 for a single node, and 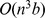 for all nodes. Let 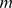 be the increased number of SNPs, and the total time complexity is 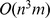.

### 2.3 Detection of Possible HREs from the Out-groups

If there are several consecutive mismatches of SNPs of a node and its parent node, it is likely that the segment is affected by some HRE. However, there might be no similar SNP segment in the given data, and we suspect it might be an HRE from an out-group. Suppose we try to assign an HRE from the out-group, since there are no known SNPs, we are free to create whatever SNPs we need to match the SNPs of the node we consider. If the weight of such HRE is a constant, it may lead to matching all the SNPs with an HRE from the out-groups. We borrow the idea of affine gap penalty in sequence alignment [12]. For the out-group HRE, we introduce the opening weight 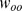 and the extending weight 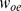. Let 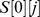 be defined the same as 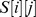 but SNP 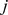 is inherited from the out-groups. The recurrence relation derived in Section 2.2 remain mostly the same except the enumeration of 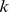 in Equation (3) and (4) should include the the out-groups. We derive the recurrence relations for the out-groups as follows.
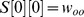


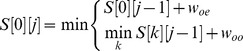



These recurrence relations can be solved by standard dynamic programming with backtracking technique, and help assign sparse mismatches as mutations/errors and dense mismatches as out-group HREs.

Sometimes the algorithm may assign two HREs of the same segment to two nodes, and they are predicted to inherit the HRE segment from each other. We consider this scenario as evidence of an out-group HRE. After assigning mutations/HREs/errors by dynamic programming and backtracking, for each SNP of a node, we trace the ancestor of the SNP allele. A SNP within an HRE segment is inherited from the HRE source, and a SNP not in an HRE segment is inherited from the parent node. If there is no HRE from out-groups, we should be able to trace all SNPs all the way to the root. If the tracing falls into a cycle, then we output the SNPs and involved nodes as evidence of an out-group HRE. This algorithm also detects inheritance patterns that form a cycle by more than two nodes.

## Experimental Results

We have implemented our algorithm in C/C++, denoted as HREfinder. We have also implemented a simulator to generate simulated data and estimate the accuracy of HREfinder. We also run HREfinder on real data obtained from SNP analysis according to [14] of all available whole genomes (draft and finished) for several bacteria and viruses.

HREfinder takes as input the SNP alleles and positional information, the genome sequences, and a phylogenetic tree. SNP detection and building a phylogenetic tree occurs prior to running HREfinder, and may be accomplished with alignment-based approaches (e.g. Mugsy [15] or progressiveMauve [16]) or the alignment free method kSNP [14], [17] (http://sourceforge.net/projects/ksnp/) which we used here with k = 25. Likewise, the method for building a phylogeny is up to the user. Here we used maximum likelihood of the SNP allele sequences [18]. The root was selected as the node that resulted in the fewest homoplastic SNPs when mapping SNPs to nodes based on alleles shared by all descendant leaves. The SNP finding and tree building methods are independent of HREfinder, but we have formatted kSNP output for automatic input to HREfinder using any of several trees (based on maximum likelihood, parsimony, neighbor joining of pairwise SNP differences, or only core SNPs).

### 3.1 Simulation

We use a model of random branching of lineages to simulate an evolutionary tree [19]. To simulate a tree of 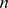 strains, we start with a root and a branching event at time 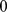. When an event occurs, it splits a lineage into two. For each new branching event, we draw a time interval from an exponential distribution with a given branching rate, then add the time interval to the current time for the occurrence time of the new event. The time interval will also be the branch length of the corresponding edge. This process stops at the time the branching event which would generate the 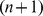
st strain is about to occur. The branch length of each edge which ends at a leaf will be assigned as the time difference between the stop time and the branching time that generated the branch. Note that the summation of the branch length on the path from the root to each leaf will be the same.

After the evolutionary tree is generated, we then need to generate genome rearrangement events. In circular bacterial genomes, inversions tend to be symmetric to the origin of replication, i.e., the endpoints of the inversion are equally distant from the origin of replication [20], [21]. Dias et al. have published a program called SIB to simulate these symmetric inversions in bacterial chromosomes [22]. We use SIB to generate inversion events. SIB generates both symmetric and nonsymmetric inversions and the number of inversions on a branch is proportional to the branch length.

After the evolutionary tree and inversion events are generated, we then generate when and on which branches mutations and HREs should occur. For each edge, we generate a series of mutation events, and the time interval between a mutation and the next mutation is drawn from the exponential distribution with a given mutation rate. The series of mutations terminates when the time of the next mutation event is later than the time of the branching event that ends the edge. For each pair of edges, consider the time interval both edges appear. In the time interval, we generate a series of HREs in the same way as described above that we generate mutations, with a given HRE rate. After all events have been generated, we uniformly randomly generate the SNPs of the root. We then generate all SNPs of all nodes in the evolutionary tree with the given mutations/HREs. The SNP position where each mutation takes place is assigned uniformly randomly. The position and length of each HRE is then generated uniformly randomly on condition that it occurs within a homologous region, i.e., the SNP order/orientation should be the same in source and destination. Finally, on the leaf nodes, we generate sequencing errors and missing loci uniformly randomly with given error rate and missing rate, respectively.

There are many HREs/mutations that cannot be detected easily, and some of them can never be detected. A mutation followed by another mutation or an HRE on the same branch will be nullified and there is no way to detect it. The SNP sequence on source and destination of an HRE may be identical or differ by only one SNP, then it has no effect or can be explained by a mutation, respectively. An HRE may be followed by another HRE on the same branch and get nullified. After simulated data is generated, we try to identify these nullified events with conditions listed above, and discard them later when computing the accuracy. By identifying and discarding nullified events in simulated data, we can compute accuracy based on events that leave some evidence. However, we only identify and discard nullifying effects that are all on the same branch when generating simulated data. We do not identify nullifying effects in which two or more branches are involved (e.g., a mutation followed by a branching event, then both branches are affected by HREs, nullifying the first mutation). Therefore, there are still some events that leave no evidence when generating simulated data, and these events will have an impact on calculating the accuracy of HREfinder. There are still many scenarios in which HREs cannot be detected: two or more HREs may overlap and can be explained by a few mutations/errors, an inversion may separate an HRE into different blocks and we cannot detect it because we consider each block separately, etc. Identifying and discarding all these events would be very difficult, and we choose not to identify all these events when generating simulated data. Therefore, many events cannot be identified as HREs, so we expect that HREfinder can detect only a subset of HREs in the simulations.

The weights of the events are set as 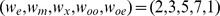. With these values, a segment that can be explained by either two (or more) mutations or one HRE from a node in the evolutionary tree, HREfinder will choose one HRE. If a segment can be explained by either three (or more) errors or one HRE from a node in the evolutionary tree, HREfinder will choose one HRE. For a segment that can be explained by one mutation or two errors, HREfinder will not explain it by an HRE. Note that the number of mutations allowed does not depend on coalescence time, since the likelihood of HREs and mutations are both proportional to the time. Our first experiment shows that most HREs are separated by inversions and cannot be detected. Therefore, in our second experiment, we do not generate inversions in order to focus on HREs within a block. The default value of parameters are: average branch length  = 20, 40 strains, 50 SNPs, mutation rate  = 1% each SNP per branch length, HRE rate  = 3% per branch length, error rate  = 1% each SNP, and missing rate = 10%. Since most HREs get partially nullified by other HREs that overlap, an HRE detected by HREfinder is considered correct if it overlaps with an actual HRE on the same branch. We denote *recall* as the number of correctly detected HREs divided by the total number of actual HREs, and *precision* as the number of correctly detected HREs divided by the total number of predicted HREs by HREfinder. The average branch length is always fixed. In each set, we try 4 different values for a parameter, and all other parameters are fixed. For each parameter setup, we run the simulation 200 times, and compute the recall and precision.

Table 3 shows the results of our simulation. A higher mutation rate brings more diversity, and it reduces the similarity between source and destination of an HRE. More diversity makes HREs easy to detect, and improves recall. However, a higher mutation rate also increases the probability of consecutive mutations, which HREfinder will explain as HRE, thus slightly decreases the precision. A higher HRE rate brings more overlapped HREs, and makes HREs difficult to detect, thus decreases the recall. A higher HRE rate also increases the precision, because it makes it easy for a detected HRE to overlap with an actual HRE. A lower missing rate results in better recall, and has little effect on the precision. The number of SNPs, or the size of a block, and the error rate, do not have significant impact on the accuracy. The number of strains can affect the accuracy either way. More strains with a fixed average branch length bring more diversity and improve the accuracy. However, more strains also bring bigger phylogenetic trees, longer simulated time, and more overlapped HREs, which lower the recall. Therefore, more strains affect recall both ways, but obviously bring a better precision.

**Table 3:** The accuracy of HREfinder under different parameters.

Mutation rate	0.5%	1%	3%	6%
Recall	31.90%	40.97%	49.07%	49.32%
Precision	79.90%	78.99%	77.33%	76.23%
HRE rate	1%	3%	6%	10%
Recall	61.58%	49.07%	38.48%	31.31%
Precision	55.32%	77.33%	85.72%	90.27%
Missing rate	1%	5%	10%	20%
Recall	51.74%	49.53%	49.07%	46.64%
Precision	77.36%	76.98%	77.33%	76.04%
Error rate	0.1%	0.5%	1%	3%
Recall	48.36%	49.35%	49.07%	48.18%
Precision	77.31%	76.66%	77.33%	76.69%
# SNPs	10	20	50	100
Recall	49.25%	48.94%	49.07%	48.51%
Precision	76.55%	76.99%	77.33%	77.04%
# strains	10	20	40	80
Recall	34.74%	48.29%	49.07%	44.61%
Precision	48.96%	63.15%	77.33%	85.81%

The default values of parameters are: average branch length  = 20, 40 strains, 50 SNPs, mutation rate  = 1% each SNP per branch length, HRE rate  = 3% per branch length, error rate  = 1% each SNP, and missing rate = 10%.

We have tried different weights of events to see how weights may affect recall and precision. The weight assignments we have tried for 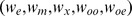 include 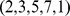, 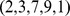, 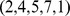, 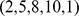, 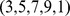. The result shows that 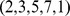 works best on recall but worst in precision. Since our objective is to find HREs, we should look for high recall rate, which is more important than high precision (low false positive), and only result for 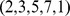 is presented here. If a user needs to reduce the number of candidate HRE’s to investigate, the weight 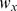 should be increased to decrease the number of false positive calls. A longer series of SNPs in a predicted HRE is more likely to be a true HRE, which could be a measure used to rank the HRE events for verification analysis.

### 3.2 Real Data

We ran HREfinder on all publicly available draft and finished genomes of *Bacillus anthracis*, *Burkholderia mallei*, *Burkholderia pseudomallei*, *Burkholderia genus*, *vaccinia virus* and *variola virus*. Nine of the B.pseudomallei genomes are in more than 1000 contigs each, so we also ran HREfinder on the subset of genomes in assembled into fewer than 100 contigs. Of the Burkholderia genus genomes, 28 were draft contigs, 11 in more than 1000 contigs. For the calculations, separate contigs or chromosomes were concatenated with 250 N’s as separators into a single sequence for each genome. Burkholderia calculations were performed on an Intel Xeon 5660 CPU with 2.8 GHz. and 48 GB RAM, and timings are given in Table 4. kSNP was run with 12 CPU, and HREfinder with 1 CPU. All data are available at https://sourceforge.net/projects/hrefinder/files/HGT_paper_data.zip. We would expect *Burkholderia mallei* and *Bacillus anthracis* to show little recombination, i.e., few HREs, and *Burkholderia pseudomallei* to show large amounts of recombination based on extensive published work [2], [3], [23], [24]. *Vaccinia virus* is also expected to show high rates of HRE resulting from a complex history due to broad host range, extensive passage in domesticated animals and chick embryos, culturing spiked with cowpox and variola, scarification practices of vaccination that reintroduced *vaccinia virus* to nature many times, and mixing of multiple vaccinia strains in vaccine preparations [25]. In contrast, *variola virus* is much more homogeneous than *vaccinia virus*, and its evolution is thought to be a result of natural selection via human-to-human transmission. As a result much lower levels of recombination have been found [26]. These in fact are the results we observe in Table 4. Most SNP discovery runs with kSNP completed in under an hour and HREfinder completed in minutes. The very large run with all public Burkholderia genomes took longer, with 69 multi-chromosome draft and finished genomes from 23 species in a 461 MB genome sequence file. The Burkholderia genus analysis probably would not be feasible for an alignment based approach for SNP discovery, unless one limited the analysis to a subset of genes such as the core genome.

**Table 4:** Summary of results from HREfinder and kSNP.

	#Genomes	Time forkSNP(h:m:ssor m:ss)	Time forHREfinder(h:m:ssor m:ss)	Total#SNPs	Homo-plasticSNPs	#coreSNPs	#blocks	# SNPsinvolvedin HRE	#HRE	# HREfromoutside
B.pseudomallei	23	55∶14	24∶13	108004	45992	45660	10168	87259	24100	331
B.pseudomallei (assembled<100 contigs)	14	30∶22	10∶29	84104	33110	46054	5395	59377	12159	456
B.mallei	11	16∶20	01∶03	1977	25	1245	256	87	15	15
Burkholderia	69	6∶28∶14	44∶56∶59	1865212	219097	38	227124	189368	33192	5130
B.anthracis	18	23∶40	00∶15	4141	57	3570	115	151	28	28
Vaccinia	33	08∶41	02∶57	3079	1564	1400	123	2818	1891	39
Variola	48	12∶56	01∶15	1725	73	1307	2	111	12	4

Core SNPs are loci present in all the genomes. Homoplastic SNPs are those that are not consistent with the pattern of inheritance in the predicted SNP-based phylogeny.

There are far too many predicted events to detail all of them, but we have looked at a subset and make the following observations, recognizing that there may be good alternative interpretations. HREfinder is intended to be used as a tool for hypothesis generation, so results are best interpreted and verified by more detailed analyses of the HRE predictions. We note that overlapping blocks result in repeatedly counting HREs, mutations, and errors, so the number of events is an overestimate that can be corrected by a user’s detailed examination of the positions and sequences of the predicted transfers. The overlapping blocks and event duplicate counting are an area for improvement of the algorithm and code. The program output includes the full sequences of the regions around the putative HRE for each of the leaf strains under the recipient node, so the region, not just the SNP allele, may be more easily compared across genomes by BLAST. For cases with many predicted HREs, increasing wx and re-running HREfinder may be required, particularly in taxa with high rates of mutation and convergent evolution.

**Figure 2:**
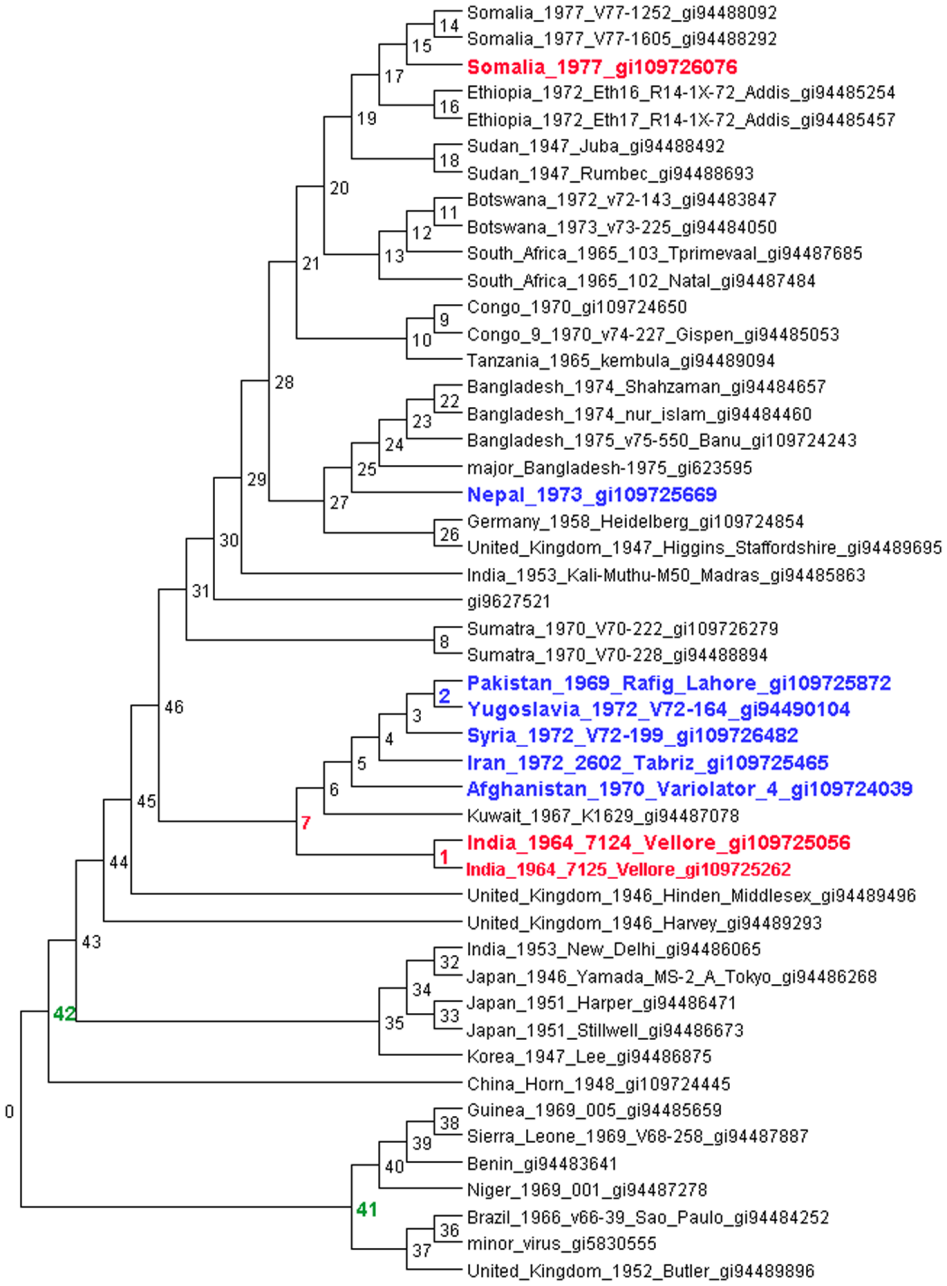
Tree for variola with node numbers indicated on internal nodes. An HRE from positions 164360–165376 is shown in red between a Somalia strain and either node 7 or node 1, although the direction of transfer is not clearly predicted by HRE. Another HRE is predicted from node 2 to the Nepal strain, shown in blue. Two more HREs are predicted from outside the tree to nodes 41 and 42, in green.

For variola, HREfinder predicts an HRE containing 16 SNPs in a sequence fragment of 1041 bases from Node 1 (the branch leading to the India 1964 Vellore strains) to the Somalia_1977_gi109726076 genome, highlighted in red in Figure 2. BLASTing the sequence from the genome corresponding to this putative HRE from positions 164360–165376 in Somalia_1977_gi109726076 shows that it is 100% identical to India_1964_7124_Vellore_gi109725056 and identical but for 6 deleted bases to India_1964_7125_Vellore_gi109725262, but only 97% identical to the other Somalia 1977 strains that are nearest neighbors. An HRE is also predicted at the same positions but in the opposite direction from Somalia_1977_gi109726076 to node 7, which is ancestral to the India 1964 strain which is identical in this region, and also branches to other Middle Eastern strains which have more indel and SNP differences in that region relative to the Somalian genome. This illustrates a circular case where HREfinder has trouble determining the direction of the event, but does suggest that an HRE in this region is possible between these regions of the tree, with convergent evolution as an alternative explanation. Genes spanned by this putative HRE region are SPI-2/CrmA IL-1 convertase, several IL-1-beta-inhibitors and hypothetical proteins, and a hypervariable AT repeat region (ABG45376.1-ABG45382.1).

Another HRE of 82 SNPs over a 6761 bp region from 17233–23969 is predicted from node 2 to the Nepal_1973_gi109725669 strain (in blue in Figure 2). This region is identical between the Nepal strain and the Pakistan, Syria, Iran, Afghanistan, and differs by only 1 base from the Yugoslavia strain, while it differs from the nearest Bangladesh strain by 8 SNPs and 1 indel. It is not clear why HREfinder predicts the HRE from node 2 instead of node 5. Genes spanned by this region are several ankyrin-like proteins, some of which are noted to inhibit NF-kB activation by preventing I-kB-alpha degradation, a SPI-3 serine protease inhibitor-like protein and an interferon resistance protein which are both noted to be host range and host defense modulators, dUTPase, a kelch-like protein, and hypothetical proteins, one of which interferes with apoptosis (AGB44808.1-ABG44817.1).

In addition to these putative HREs within the tree, 2 events are predicted from outside the tree, shown in green on Figure 2. One is predicted to node 41, which branches to the variola minor strains and spans 5 SNPs from positions 171335–171464 (relative to strain Bangladesh_1974_nur_islam_gi94484460). The other, to node 42, branches to the variola major strains, and spans positions 72847–74129. The first region spans mostly an intergenic region and the beginning of a kelch-like protein (ABF23763.1), and the second begins on a polyA polymerase subunit (ABF23656.1)and ends on a DNA-dependent RNA polymerase subunit rpo22 (ABF23657.1). Note that these positions are the SNPs closest to the ends of the putatively transferred region, and the end of the HRE could extend beyond.

The only HREs predicted in *B. anthracis* are from “outside” the tree, the majority to internal nodes. The majority are predicted to the node branching to A0442 and Kruger_B, as well as some to the leaf node A1055 and one to A0488. This indicates that the affected node differs from other nodes in the tree by a series of co-linear SNPs. This could result from HRE from an unsequenced isolate, or it could result from positive selection in a particular region which changes a series of SNPs, and suggest that more detailed analyses are needed for these regions. BLASTing the *B. anthracis* putative HRE regions from leaf nodes against all Bacillaceae genomes show one region (strain A1055_positions 2496911–2503816) with highest similarity to Bacillus thuringiensis serovar andalousiensis BGSC 4AW1 and other regions with highest similarity to proprietary, unpublished draft isolates sequenced by collaborators.

For vaccinia, all of the HREs predicted by HREfinder from outside the tree are to Vaccinia_Horsepox_virusMNR-76_gi111184167. One of these putative HRE regions spans positions 211–1425 and has the top BLAST hit to monkeypox Zaire (gi|17529780). Another very large putative HRE from positions 88078–106214 in Tian Tan is predicted to come from within the tree from the WR strain (Vaccinia_gi66275797, and indeed this is the top BLAST hit for the region, a better match than the more closely related Copenhagen and rabbitpox strains. There are also events predicted between Dryvax clones. One putative HRE from the WR strain to Vaccinia_GLV-1h68_gi167412463 positions 81817–91609 actually has the best BLAST matches to Homo sapiens transferrin receptor, so appears to be a region that is involved in HRE not only among vaccinia, but between virus and host.

HREfinder predicts very few HREs in *B. mallei*. The node branching to NCTC_10229, NCTC_10247, and 2002721280 has the majority of predicted transfers which come from outside the tree. The longest includes only 16 SNPs, and most are much shorter.

**Figure 3:**
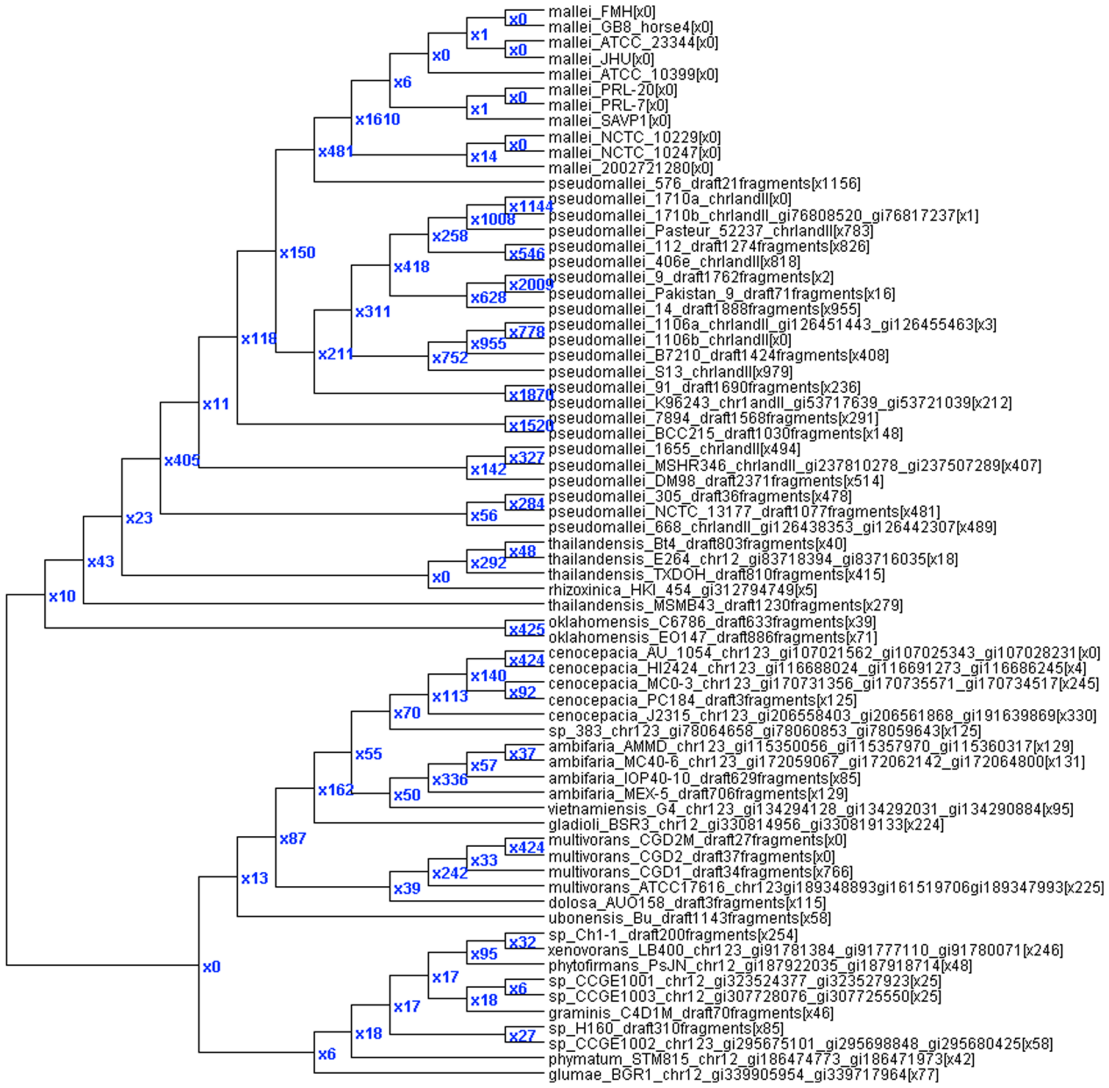
SNP tree for Burkholderia with putative HRE counts. This tree shows the number of predicted HREs (x#) to each node and to each genome in brackets after the genome name.

We analyzed *B. pseudomallei* both with and without highly fragmented draft genomes. Including the 9 additional draft genomes resulted in more SNP loci, although slightly fewer core SNP loci present in all genomes, some of these possibly due to gaps and errors that obscure the locus in highly fragmented drafts. There are almost twice the number of blocks and HREs when the extra draft genomes are included, but only 50% more SNPs predicted to be involved in HREs, since the larger number of blocks breaks up HREs into more, smaller putative transfers. However, fewer HREs are predicted from outside the tree when the additional draft genomes are included, supporting the hypothesis that HREs from unsequenced isolates can parsimoniously explain a series of novel SNP alleles. Finally, the analyses of 69 highly divergent genomes from the Burkholderia genus (Figure 3) illustrates several points: 1) pseudomallei has far more putative HREs than other species; 2) mallei has by far the fewest predicted HREs; 3) other species that cluster separately from the (mallei, pseudomallei, rhizonica, thailandensis, oklahomensis) cluster appear to have intermediate levels of HREs between mallei and pseudomallei. In the node leading exclusively to the mallei strains, 1610 HREs are predicted, only 8 from outside the tree, 197 from pseudomallei 668, 221 from the node leading to pseudomallei 9 and Pakistan 9, 118 from the node leading to pseudomallei 1710a and 1710b, 103 from the node leading to pseudomallei 1106a and 1106b, and dozens from other pseudomallei internal and leaf nodes.

**Figure 4:**
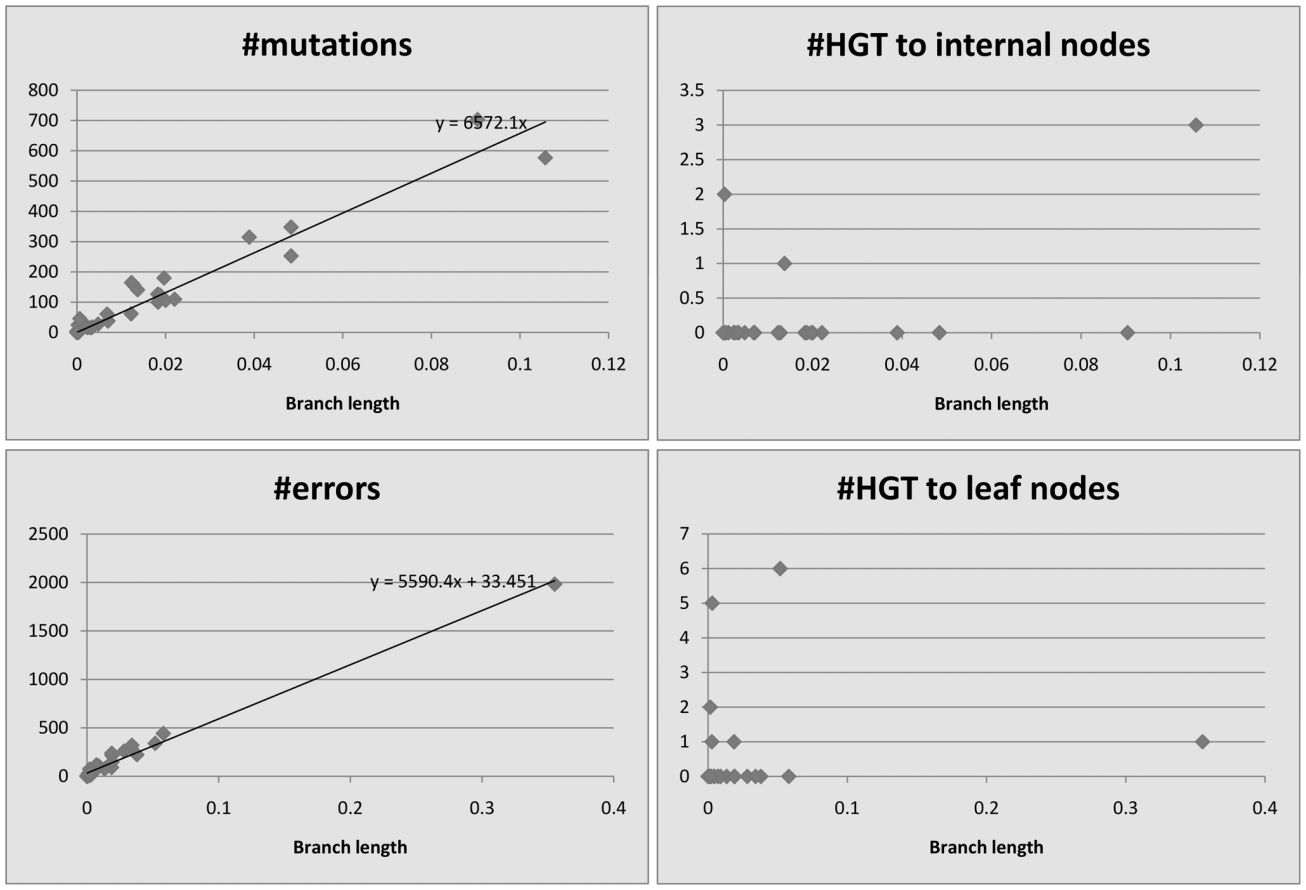
*Bacillus anthracis*, 34 strains, 8781 SNPs (increased).

**Figure 5:**
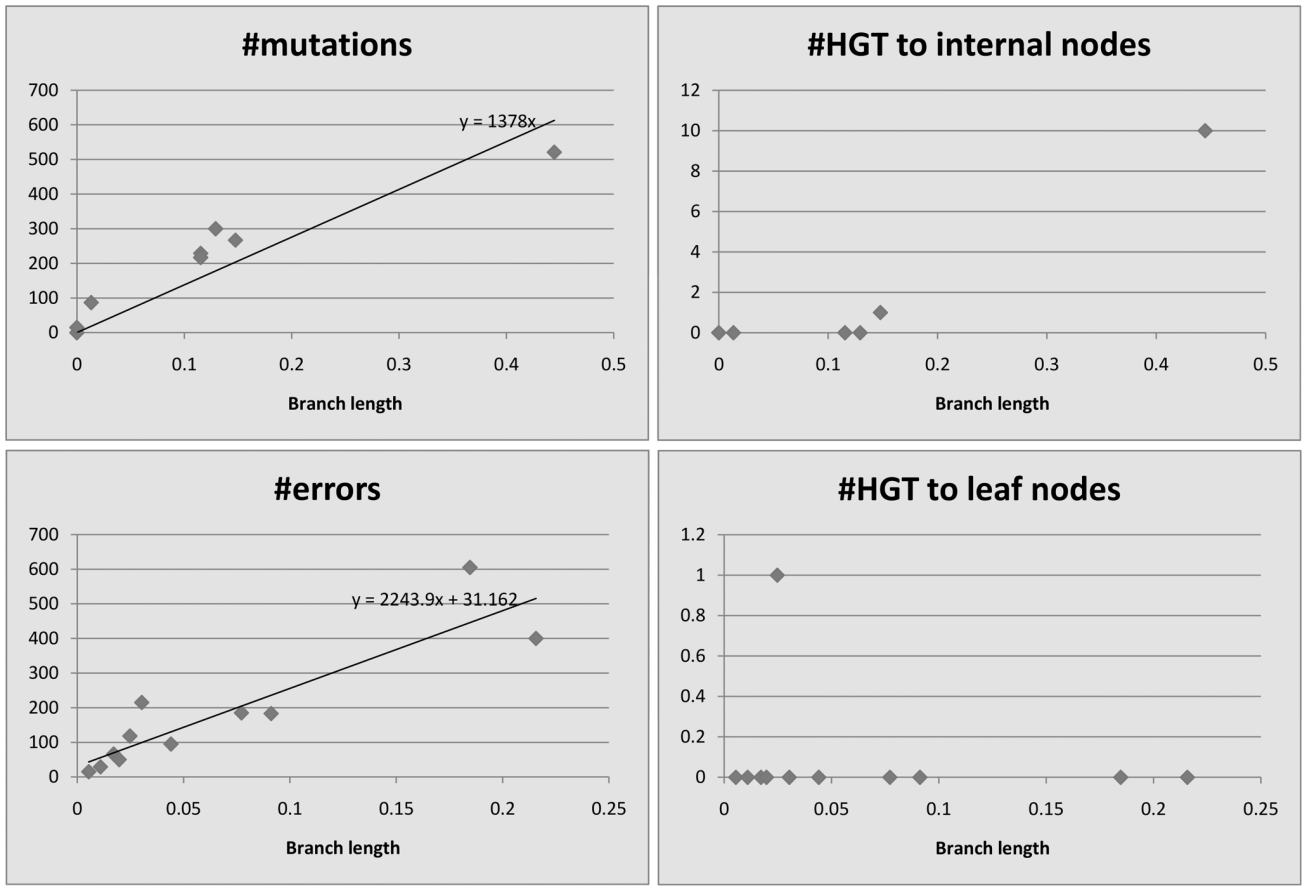
*Burkholderia mallei*, 11 strains, 3659 SNPs (increased).

**Figure 6:**
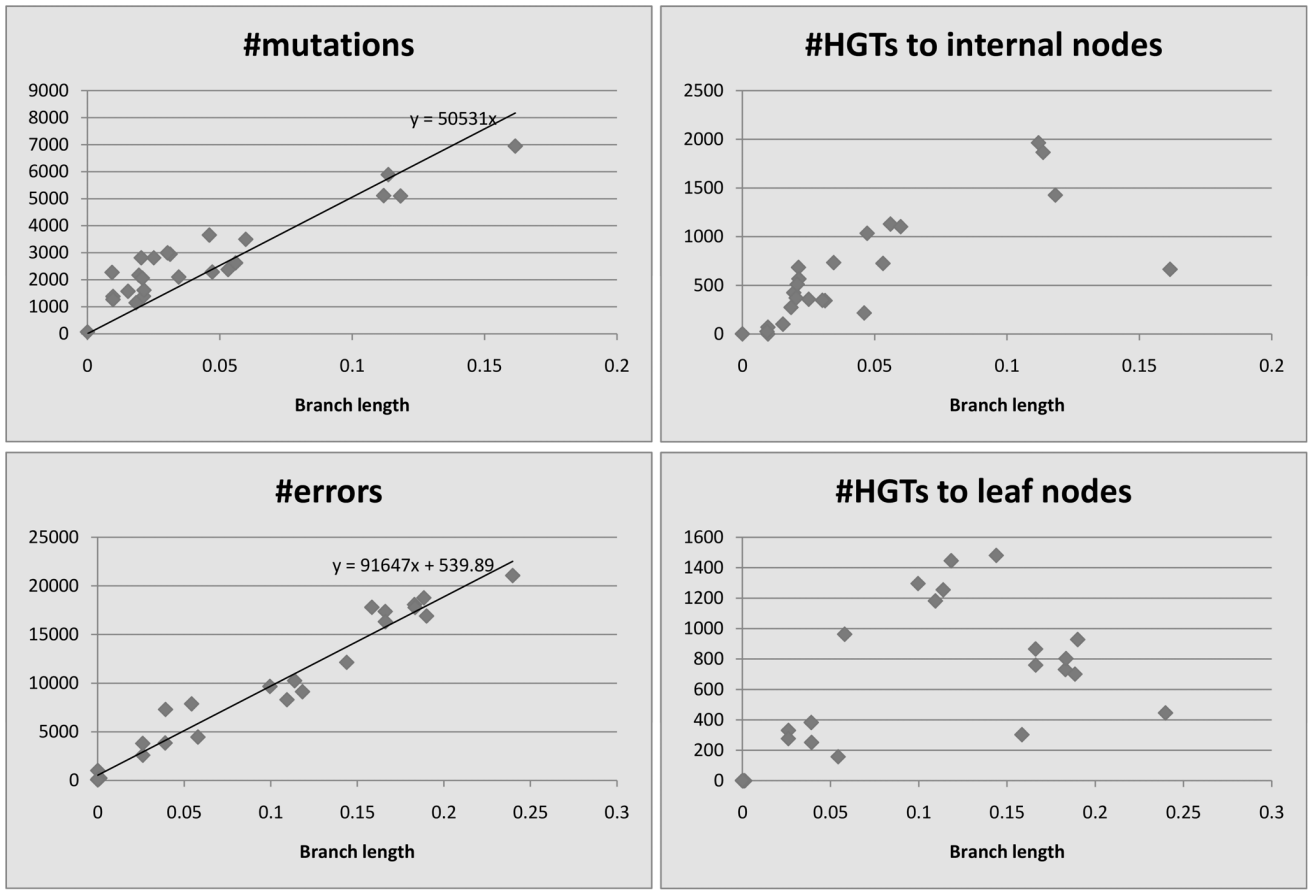
*Burkholderia pseudomallei*, 26 strains, 212174 SNPs (increased).

**Figure 7:**
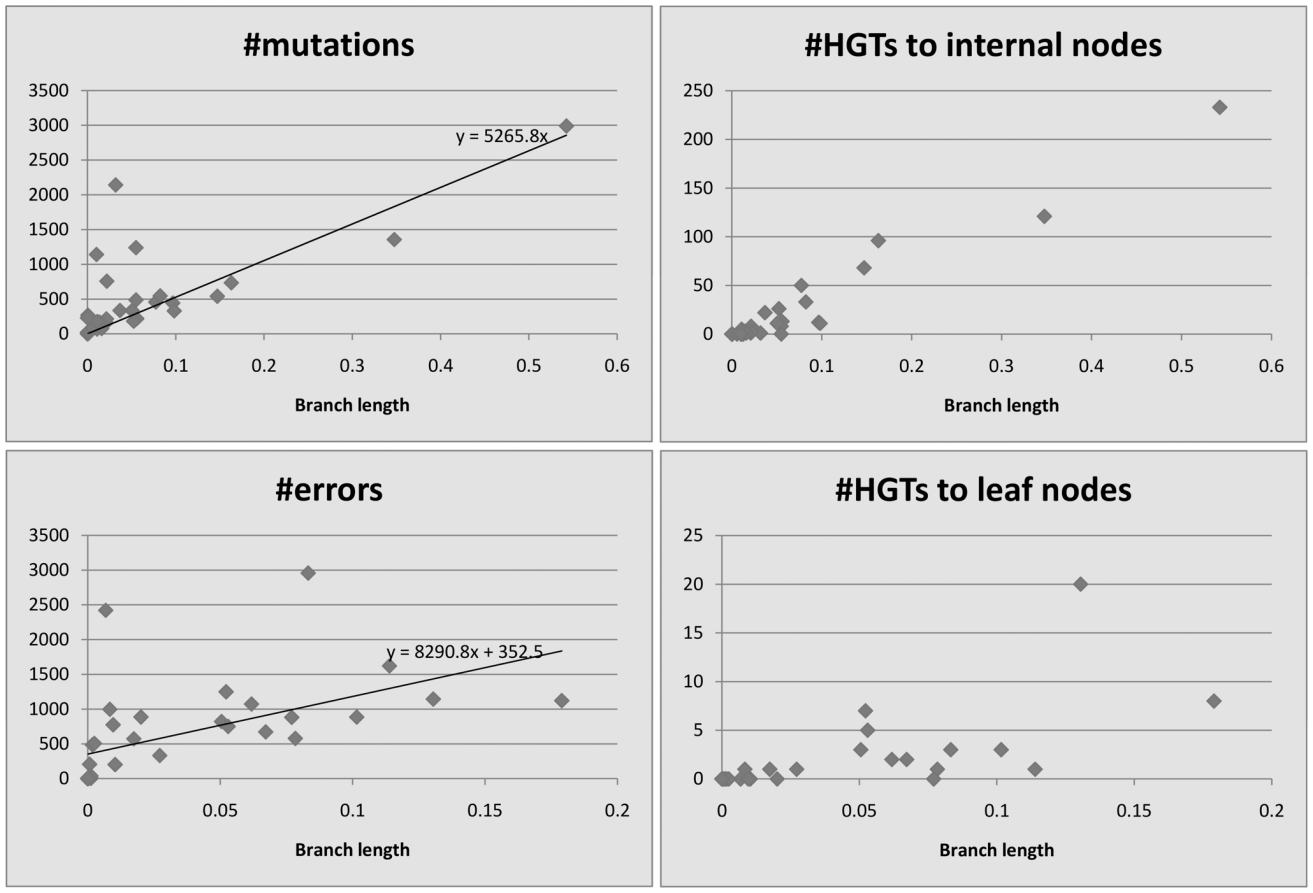
*Vaccinia virus*, 33 strains, 17562 SNPs (increased).

**Figure 8:**
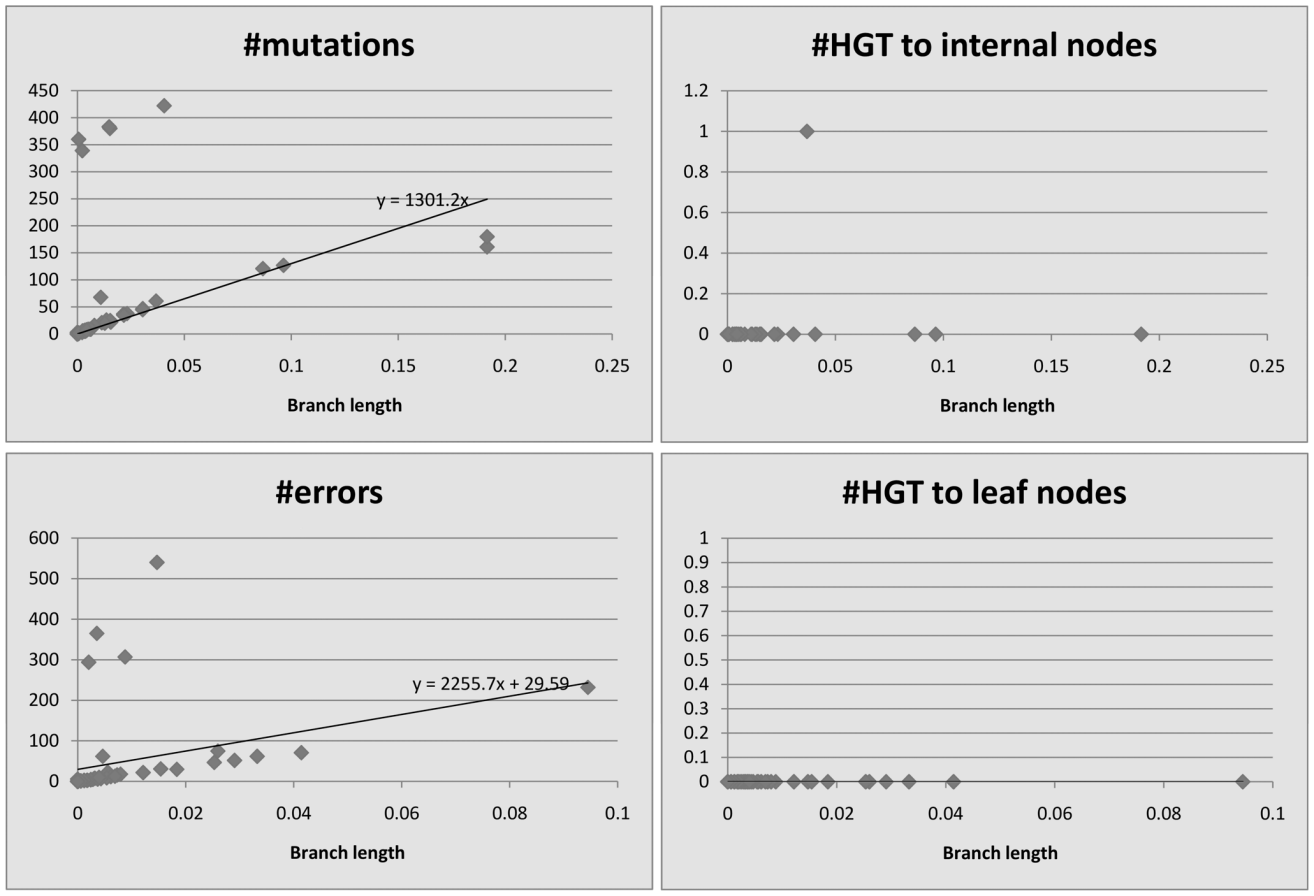
*Variola virus*, 49 strains, 2926 SNPs (increased).

We plot the number of events detected by HREfinder as a function of the length of the branches. Figures 4, 5, 6, 7 and 8 show the results. Note that blocks may overlap heavily because of duplications, so that some SNPs may be computed many times, increasing the count of mutations. Therefore we use the increased number of SNPs as a reference of mutation counts. The branch length is calculated by the rearrangement distance, which we expect to be proportional to the evolutionary time [17]. We also expect the number of mutations to be proportional to the evolutionary time, thus proportional to the branch length, which is consistent with the plots. For leaf nodes, mutations are considered as errors. The number of errors should only be proportional to the number of SNPs. If we draw a linear trendline 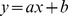 where 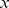 is the branch length and 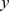 the number of errors and mutations, then the intercept 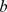 should represent the number of errors. Given the intercepts are small in our plots, most “errors” on the leaf nodes should be mutations. The difference of slopes between mutations and errors in the plots could be because the accuracy of branch length estimation is different between internal nodes and leaf nodes. A few branches are outliers, however, showing more mutations than expected based on the branch length, which could be explained by the following. In *Burkholderia mallei* dataset, there are many blocks that overlap extremely heavily, and mutations in the overlapping regions get counted repeatedly. In the other 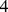 datasets, there are some regions that get partitioned into many single-SNP blocks by HREfinder, and some HREs fall into these regions. HREfinder explains these HREs by many mutations or errors, and it leads to some plots with extreme amounts of mutations and errors.

For homogeneous species like *Bacillus anthracis*, *Burkholderia mallei* and *variola virus*, there appears to be no relationship between HREs and branch length, since so few HREs have occurred. Even for the more heterogeneous *Burkholderia pseudomallei* and *vaccinia virus*, HREs seem to have much weaker relationship to branch length than do mutations or errors. HRE may have less to do with evolutionary time (branch length) and more to do with ecological opportunity. Factors like co-infection or co-habitation in the environment with multiple strains or species could lead to more opportunities for HRE, as could the prevalence of genetic mechanisms for HRE like transposons or other mobile elements. There are more HREs to internal nodes than to leaf nodes. We believe it is because the weight of an error is smaller than that of a mutation, so HREfinder with the weight parameter settings used here tends to assign errors on leaf nodes but HREs on internal nodes.

**Figure 9:**
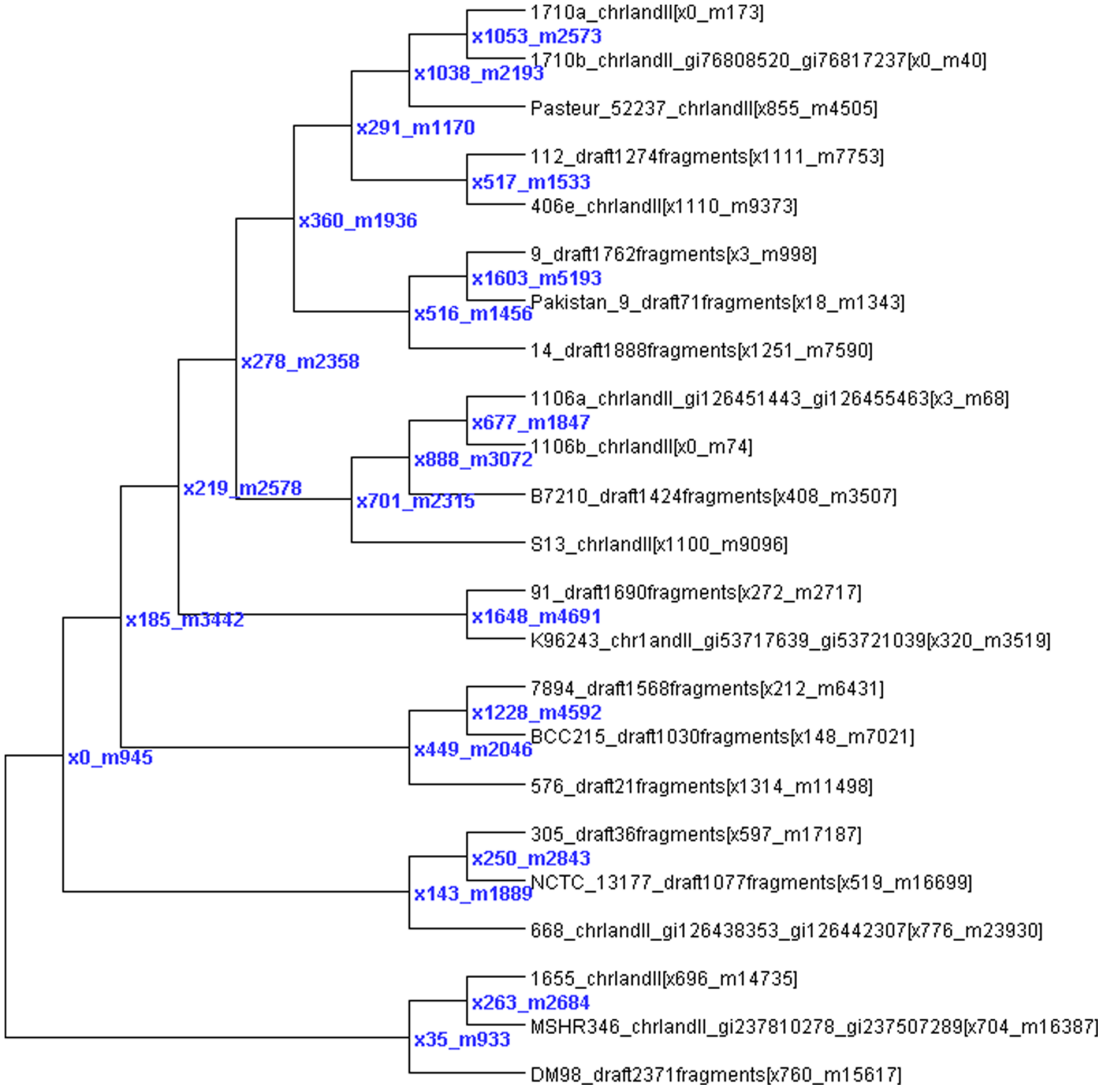
Evolutionary tree of *Burkholderia pseudomallei*. The internal nodes are labeled by the number of events, m for mutation, e for error, and x for HRE.

**Figure 10:**
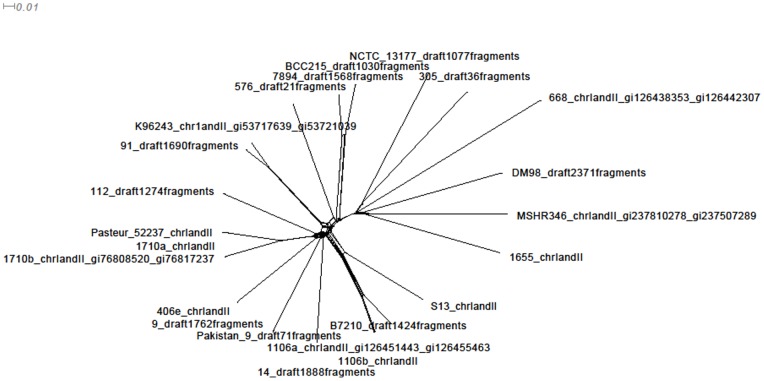
Phylogenetic network generated using SplitsTree v4 based on the SNP alleles for *Burkholderia pseudomallei*.

We plot the number of mutation/HRE/error events of *Burkholderia pseudomallei* dataset in Figure 9 with Dendroscope [27] and outline the number of HREs from the out-groups of each strain. We also show a phylogenetic network (Figure 10) created from the SNP data using SplitsTree [28], which illustrates the reticulate nature of the tree but does not easily allow us to show predicted numbers mutations and HREs. There are 331 out of 24100 HREs predicted to be from out-groups and 240 of them are on the leaf nodes. HREfinder outputs the full sequence of HRE regions from leaf nodes (including the sequence between SNPs). BLASTing HRE regions in *Burkholderia pseudomallei* that are predicted to come from out-groups shows many with high homology to transposon, phage, and plasmid sequences, which are prime candidates for HREs. Others show strong homology to soil and water inhabiting microbes like Rhizobium, Pseudomonas, and other Burkholderia species, consistent with HREs occurring in soil and aquatic environments.

## Conclusions

We designed and implemented an algorithm to do HRE detection among many whole genomes using dynamic programming, based on SNPs. Our experimental results on simulated data show that there are many HREs that cannot be detected, but the HREs detected by our program are mostly true events. The tradeoff between recall and precision depend on the weights used, so a user may modify depending on tolerance for false positives/negatives. HREfinder is intended for hypothesis generation, and should be followed up by more detailed analyses of sequences, not just SNPs, to verify predicted HREs. The experimental results on real sequence data show that the number of HREs we predict for several bacteria and viruses is consistent with expectations based on the literature, and BLAST similarity of some of the putatively transferred regions support the predictions of HREfinder.
